# Mitochondrial Adaptations to Oxidative Stress Confer Resistance to Apoptosis in Lymphoma Cells

**DOI:** 10.3390/ijms130810212

**Published:** 2012-08-16

**Authors:** Sarah T. Wilkinson, Margaret E. Tome, Margaret M. Briehl

**Affiliations:** Department of Pathology, University of Arizona, Tucson, AZ 85724-5043, USA; E-Mails: stw@email.arizona.edu (S.T.W.); mtome@pathology.arizona.edu (M.E.T.)

**Keywords:** mitochondria, cancer, reactive oxygen species, catalase, cytochrome *c*, cardiolipin

## Abstract

Acquired resistance to drugs commonly used for lymphoma treatment poses a significant barrier to improving lymphoma patient survival. Previous work with a lymphoma tissue culture model indicates that selection for resistance to oxidative stress confers resistance to chemotherapy-induced apoptosis. This suggests that adaptation to chronic oxidative stress can contribute to chemoresistance seen in lymphoma patients. Oxidative stress-resistant WEHI7.2 cell variants in a lymphoma tissue culture model exhibit a range of apoptosis sensitivities. We exploited this phenotype to test for mitochondrial changes affecting sensitivity to apoptosis in cells made resistant to oxidative stress. We identified impaired release of cytochrome *c*, and the intermembrane proteins adenylate kinase 2 and Smac/DIABLO, indicating inhibition of the pathway leading to permeabilization of the outer mitochondrial membrane. Blunting of a glucocorticoid-induced signal and intrinsic mitochondrial resistance to cytochrome *c* release contributed to both points of resistance. The level of Bcl-2 family members or a difference in Bim induction were not contributing factors. The extent of cardiolipin oxidation following dexamethasone treatment, however, did correlate with apoptosis resistance. The differences found in the variants were all proportionate to the degree of resistance to glucocorticoid treatment. We conclude that tolerance to oxidative stress leads to mitochondrial changes that confer resistance to apoptosis.

## 1. Introduction

Non-Hodgkin lymphoma is commonly associated with chronic infection and chronic inflammation [1]. Chronic inflammation has been recognized as an enabling characteristic in the development of cancer [2]. Such conditions are often characterized by chronic oxidative stress, itself considered a risk factor for the development of cancer [3]. However, the mechanism by which chronic oxidative stress promotes cancer has yet to be fully elucidated. One possibility is that it selects for resistance to apoptosis, which is one of the hallmarks of cancer [2]. This phenotype could allow for cell transformation while also conferring treatment resistance. Since a number of anticancer agents work at least in part by increasing cellular oxidative stress [4], chronic inflammation may pose a particular risk for the development of treatment-refractory cancers.

Oxidants are known inducers of apoptosis. Like many chemotherapeutic agents, including glucocorticoids, oxidants can work through the mitochondrial (intrinsic) pathway to apoptosis [5–8]. In this pathway, apoptotic signals lead to permeabilization of the outer mitochondrial membrane and release of cytochrome *c* and other apoptotic factors into the cytosol, with the subsequent degradation of key cellular components [9]. Cytochrome *c* release marks the transition from the signaling phase to the execution phase of apoptosis and is thought to occur by a two-step process [10–12]. Although some fraction of the cytochrome *c* population is soluble in the intermembrane space, the majority is localized to the outer leaflet of the inner mitochondrial membrane through electrostatic interactions with the electron transport chain and the phospholipid cardiolipin. When cardiolipin becomes oxidized, cytochrome *c* is freed into the intermembrane space as a soluble protein, the first step toward release [11,12]. The second step involves mitochondrial outer membrane permeabilization (MOMP) by the Bcl-2 family and the release of cytochrome *c* and other already-soluble apoptogenic factors into the cytosol [12,13]. The antagonistic interplay of the Bcl-2 family controls MOMP. Oligomerization of Bak and/or Bax forms a pore in the outer membrane [14]. BH3-only family members, e.g., Bim, tBid, initiate or promote pore formation while anti-apoptotic Bcl-2 family members, e.g., Bcl-2, Bcl-X_L_, Mcl-1, inhibit it [15,16].

Our previous work with an *in vitro* lymphoma model has demonstrated that selection for resistance to oxidative stress confers concurrent resistance to chemotherapy-induced apoptosis [17–19]. The lymphoma model system consists of the WEHI7.2 murine thymic lymphoma-derived cell line and variants established by transfection of these cells with catalase (CAT2, CAT38) or by gradual selection for growth in the presence of hydrogen peroxide (200R) [17,18]. Compared to WEHI7.2 cells and control transfectants, these oxidative stress-resistant variants demonstrate delayed release of cytochrome *c* and a significant inhibition of apoptosis following treatment with various chemotherapy-inducing agents used to treat lymphoma, including glucocorticoids [17–19].

The delayed cytochrome *c* release seen following dexamethasone treatment of the oxidative stress-resistant lymphoma cell variants indicates that the mechanism of apoptosis resistance lies upstream of this event. We know that signaling in response to glucocorticoids has been altered in the oxidative stress-resistant variants. While there is no difference in the generation of hydrogen peroxide, a key signal necessary for undergoing apoptosis, they do demonstrate an increased removal of hydrogen peroxide that is proportionate to catalase (over-)expression [20]. Mitochondria are central to the decision to undergo apoptosis in response to oxidant signals, in particular, by regulating the release of cytochrome *c* [21,22]. Recent work by Letai and colleagues shows that the propensity of tumor cells to undergo mitochondrially-mediated apoptosis correlates with clinical response to chemotherapy [23]. This suggests that mitochondrial alterations in cells resistant to oxidative stress could influence drug response in the clinic. Here, we have tested for mitochondrial changes affecting sensitivity to drug-induced apoptosis in the lymphoma cells made resistant to oxidative stress. In particular, we have focused on mitochondrial determinants of cytochrome *c* release.

## 2. Results and Discussion

### 2.1. Oxidative Stress-Resistant Lymphoma Cells Have Apoptosis-Resistant Mitochondria

The oxidative stress-resistant lymphoma variants that we established show a range of sensitivities to apoptosis induced by glucocorticoids and other chemotherapeutic agents [17–19]. This range makes it feasible to identify mitochondrial alterations that correspond with apoptosis sensitivity. By 24 h of treatment with the synthetic glucocorticoid dexamethasone, the WEHI7.2 and control transfectant cells begin to release cytochrome *c* and undergo apoptosis. In contrast, cytochrome *c* release is not seen in 200R and CAT38 cells until 32 h of dexamethasone treatment, and dexamethasone treatment for up to 40 h fails to release cytochrome *c* in CAT2 cells [17,18]. In the current study, we included Hb12 cells as a positive control for a mitochondrial change known to confer resistance to apoptosis. These cells were established by stable transfection of WEHI7.2 cells with *bcl-2* [24]. Similar to CAT2 cells, Hb12 cells show no release of cytochrome *c* after 40 h of dexamethasone treatment.

We used a cell-free apoptosis assay [25,26] to compare the functional ability of WEHI7.2 parental and oxidative stress-resistant variants to release cytochrome *c*. Cells were treated with 1 μM dexamethasone or vehicle control; a 12 h time point was chosen to put the cells in the signaling phase of apoptosis, upstream of cytochrome *c* release in whole cells. The parental WEHI7.2 cells act as a control for 200R cells, while NEO control-transfectant cells act as a control for the CAT2, CAT38, and Hb12 cells. Mitochondria were isolated and incubated with purified, recombinant tBid, a pro-apoptotic protein known to trigger mitochondrial outer membrane permeabilization.

**Figure 1:**
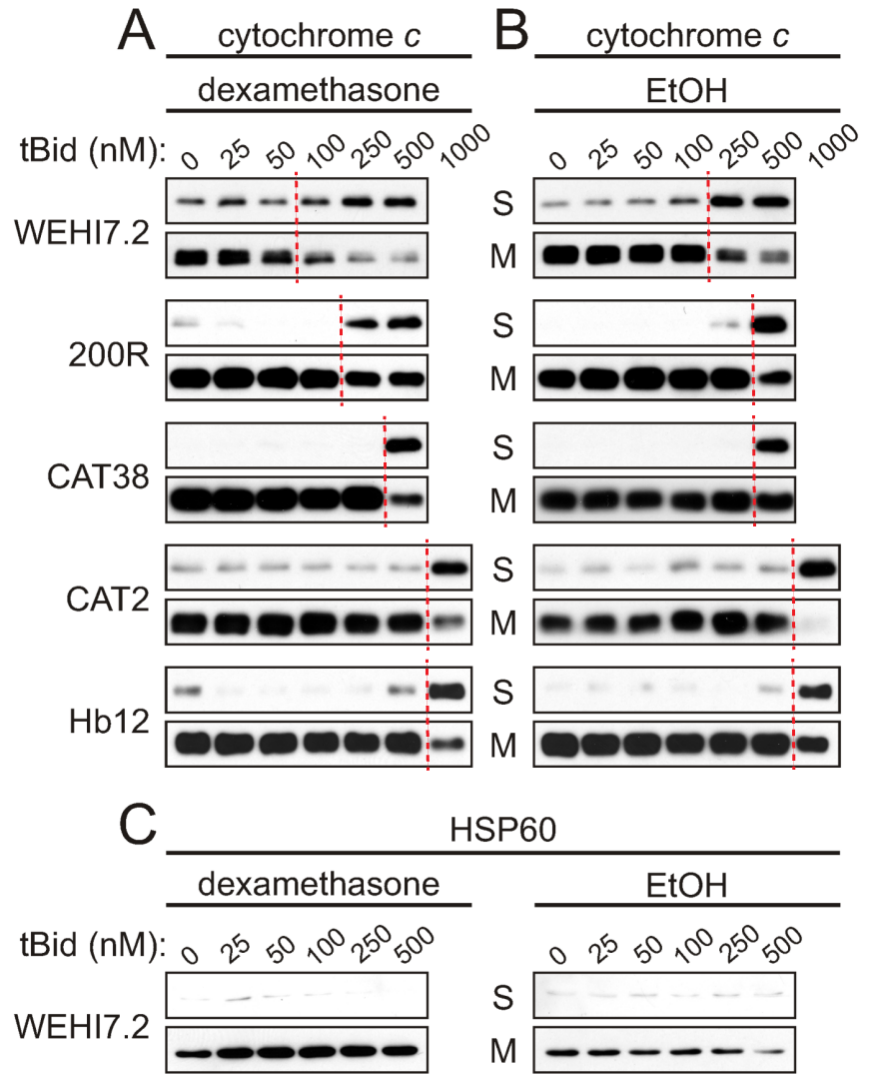
tBid-induced cytochrome *c* release is inhibited in mitochondria from oxidative stress-resistant WEHI7.2 lymphoma variants. WEHI7.2 parental, hydrogen peroxide-resistant (200R), catalase transfected (CAT38, CAT2), and Bcl-2 transfected (Hb12) cells were pre-treated with 1 μM dexamethasone (**A**) or EtOH vehicle (**B**) for 12 h. Mitochondria were isolated, treated with various concentrations of recombinant tBid, and separated into mitochondrial (M) and supernatant (S) fractions. Dashed lines indicate where marked cytochrome *c* release from the mitochondria to the supernatant fractions is first apparent. Representative immunoblots for cytochrome *c* (**A** and **B**) and HSP60 (**C**) from 3–10 independent experiments for each cell type are shown. HSP60 is a mitochondrial matrix protein used as a control for the mitochondrial preparation and equivalent protein loading of samples.

The results show that glucocorticoid treatment sensitizes WEHI7.2 mitochondria for cytochrome *c* release (Figure 1). A concentration-dependent release of cytochrome *c* in response to tBid was seen for mitochondria from both the dexamethasone- and vehicle-treated WEHI7.2 cells, however, release from dexamethasone-treated cells’ mitochondria occurs with less tBid. Dashed lines in Figure 1 indicate the point at which a marked loss of cytochrome *c* in the mitochondrial fraction, and resultant gain in the supernatant fraction, become apparent. For dexamethasone-treated cells’ mitochondria, cytochrome *c* release occurs with more than 50 nM tBid (Figure 1A), while mitochondria from vehicle-treated cells (Figure 1B) require more than 100 nM tBid to cause release. Vector-transfected NEO cells demonstrated similar kinetics of cytochrome *c* release to WEHI7.2 cells (Supplementary Figure S1). Control experiments for the mitochondrial matrix protein, HSP60, confirmed that the integrity of the inner mitochondrial membrane was maintained during the isolation procedure (Figure 1C and Supplementary Figures S1 and S2).

If the oxidative stress-resistant cells are removing a dexamethasone-induced signal generated upstream of the mitochondria, which sensitizes the mitochondria for cytochrome *c* release, we expect to see no difference in sensitivity to tBid when comparing cytochrome *c* release in the vehicle-treated and dexamethasone-treated samples of a given variant. Dexamethasone treatment only slightly enhanced tBid-induced cytochrome *c* release from 200R and CAT38 mitochondria, and did not increase the sensitivity in the CAT2 and Hb12 cells (compare Figure 1A and 1B). Indeed, the mitochondria from dexamethasone-treated CAT2 cells were even more resistant to tBid-induced release than mitochondria from vehicle-treated CAT2 cells. Thus, selection for resistance to oxidative stress has blunted a dexamethasone-induced signal for cytochrome *c* release. These results are proportionate to the removal of hydrogen peroxide in the apoptosis-resistant variants [20].

We also observed an intrinsic resistance to tBid-induced cytochrome *c* release in mitochondria isolated from the variants. This was evident from the higher tBid concentrations needed to release cytochrome *c* from mitochondria of the vehicle-only treated oxidative stress-resistant cells as compared to WEHI7.2 cells (Figure 1B). A tBid concentration of 1000 nM was needed to release cytochrome *c* from mitochondria of the most resistant variants, CAT2 and Hb12 cells, as compared to 250 nM tBid needed for release from WEHI7.2 mitochondria. An intermediate concentration was needed for the 200R and CAT38 cells. Thus, selection for resistance to oxidative stress appears to have blunted mitochondrial priming for cytochrome *c* release in the variants.

### 2.2. tBid-Induced Release of Mitochondrial Intermembrane Space Proteins Is Attenuated in the Oxidative Stress-Resistant Cells

Although the above experiments demonstrated that cytochrome *c* release was inhibited in the oxidative stress-resistant variants, they do not offer insight as to which step of cytochrome *c* release was being affected. Our next experiments investigated the extent to which the inhibition of cytochrome *c* release from mitochondria of oxidative stress-resistant cells was due to altered outer membrane permeability. As described in the Introduction, cytochrome *c* release is thought to proceed via a two-step process: oxidation of cardiolipin to solubilize it in the intermembrane space, followed by permeabilization of the outer mitochondrial membrane by the Bcl-2 family. In contrast, adenylate kinase 2 (AK-2) and Smac/DIABLO are soluble intermembrane space proteins that require only the permeabilization of the outer mitochondrial membrane by Bcl-2 proteins to be released, and do not interact electrostatically with mitochondrial membrane components, *i.e.*, cardiolipin [12,27].

**Figure 2:**
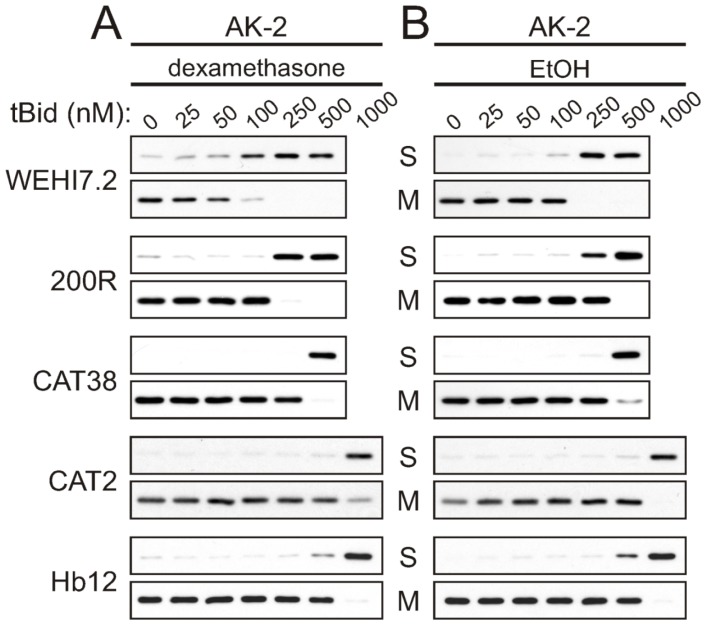
tBid-induced AK-2 release is inhibited in mitochondria from oxidative stress-resistant WEHI7.2 lymphoma variants. WEHI7.2 parental, 200R, CAT38, CAT2, and Hb12 cells were pre-treated with 1 μM dexamethasone (**A**) or EtOH vehicle (**B**) for 12 h. Mitochondria were isolated, treated with various concentrations of recombinant tBid, and separated into mitochondrial (M) and supernatant (S) fractions. Representative immunoblots from 3–10 independent experiments for each cell type are shown.

To determine whether altered outer membrane permeability was limiting cytochrome *c* release in the variants, we used the cell-free apoptosis assay to test for tBid-induced release of AK-2 and Smac. If there were no differences in control of mitochondrial outer membrane permeability, tBid would be expected to induce release of AK-2 and Smac/DIABLO as readily in the resistant variants as in WEHI7.2 parental cells. The results show that following treatment with dexamethasone or vehicle, a higher concentration of tBid was required for AK-2 (Figure 2) and Smac (Supplementary Figure S3) release from the resistant cells’ mitochondria, as compared to mitochondria from WEHI7.2 and NEO cells. As with cytochrome *c* release, the 200R and CAT38 mitochondria showed intermediate resistance while the CAT2 and Hb12 mitochondria were the most resistant to AK-2 and Smac release in response to tBid. Dexamethasone treatment was once again seen to sensitize mitochondria to tBid-induced release of the intermembrane space proteins, with the greatest effect in WEHI7.2, followed by 200R and CAT38 cells, and no sensitization in the CAT2 variants. Thus, the pattern of resistance to tBid-induced release of AK-2 and Smac/DIABLO parallels that of resistance to cytochrome *c* release. An additional finding from these experiments was that release of AK-2 and Smac, once it occurred, was more complete than what had been seen for cytochrome *c* release (compare Figures 1 and 2). This was observed for both the WEHI7.2 cells and the variants.

### 2.3. Altered Permeability of the Outer Mitochondrial Membrane Is Not due to Differences in the Ratio of Bcl-2 Family Members or Induction of Pro-Apoptotic Bim

**Figure 3:**
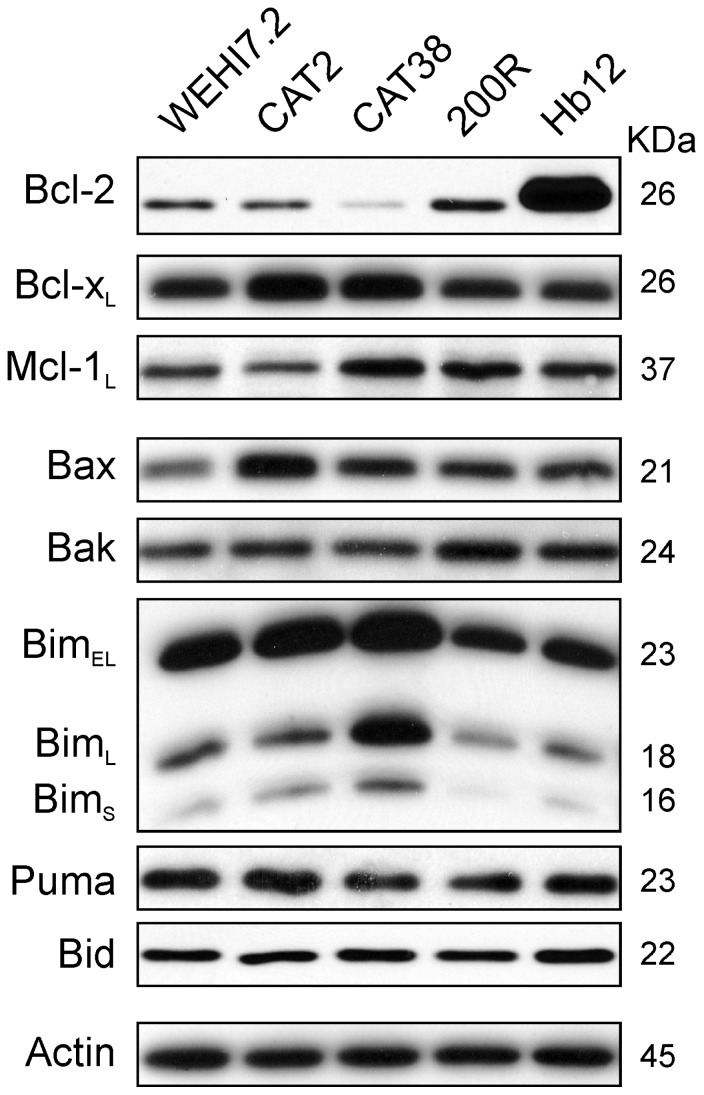
Basal expression of Bcl-2 family proteins does not explain the resistance of the variant cells’ mitochondria to apoptosis. Immunoblots of whole cell lysates (25 μg) from untreated WEHI7.2 parental, CAT2, CAT38, 200R, and Hb12 cells were probed with antibodies to the indicated Bcl-2 family members. Representative immunoblots from a minimum of three independent experiments are shown. A representative actin blot is shown as a loading control.

Selection for resistance to oxidative stress could have changed the level of Bcl-2 family members, thereby altering the permeability of the outer mitochondrial membrane. An increase in the ratio of anti-apoptotic to pro-apoptotic Bcl-2 family members can cause apoptosis resistance [28] by regulating the permeability of the outer mitochondrial membrane (reviewed in [15]). Bcl-2, Bcl-x_L_ and Mcl-1_L_ function as key anti-apoptotic proteins in lymphoid cells, while key pro-apoptotic family members are Bax, Bak, Bim, Puma and Bid [16]. A comparison of the levels of these proteins in untreated WEHI7.2 *vs.* 200R, CAT38 and CAT2 cells, however, did not show a relative increase in the anti-apoptotic to pro-apoptotic members that would explain the apoptosis resistance (Figure 3). As expected, increased Bcl-2 levels were seen in the Hb12 cells used as a positive control. Small increases in an anti-apoptotic protein seen in an oxidative stress-resistant variant were balanced by an increase in a pro-apoptotic family member (e.g., Bcl-x_L_ and Bax in CAT2 cells). Thus, basal levels of these proteins do not account for the blunted priming of the variants’ mitochondria to apoptosis.

**Figure 4:**
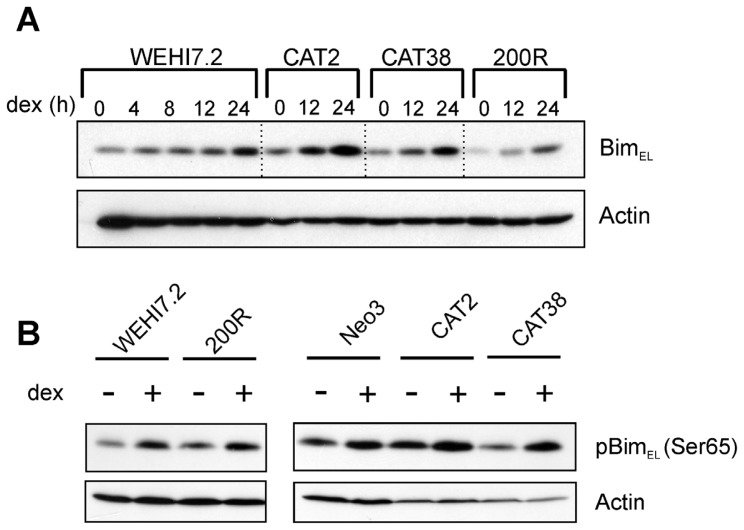
Dexamethasone-induced Bim expression (**A**) and phosphorylation (**B**) do not explain the resistance of the variant cells’ mitochondria to apoptosis. Immunoblots of whole cell lysates (100 μg) from WEHI7.2 parental, CAT2, CAT38, 200R, and Hb12 cells pre-treated with 1 μM dexamethasone for 12 h were probed with indicated antibodies. Representative immunoblots from a minimum of three independent experiments are shown; a representative actin blot is shown as a loading control.

Although basal levels of Bcl-2 family members did not explain resistance, we hypothesized that induction of the BH3-only pro-apoptotic protein Bim in response to apoptosis induced by dexamethasone might vary between sensitive and resistant cells. Bim induction has been demonstrated at the mRNA and protein level in response to dexamethasone [29] and is an essential step in apoptosis [29–31]. We anticipated that this increase in Bim would be absent in apoptosis-resistant CAT2, CAT38, and 200R variant cells treated with 1 μM dexamethasone. Figure 4A shows a time-course of glucocorticoid-induced Bim_EL_ expression. As expected, Bim_EL_ was induced in WEHI7.2 parental cells, as demonstrated previously [29], but it was also induced in the CAT2, CAT38, and 200R resistant variants. Post-translational modifications of Bim are correlated with its localization and apoptotic potency [32–34]. Phosphorylation of Bim in response to several agents, including glucocorticoids, is part of the pro-apoptotic signaling pathway [30,35]. It is possible that phosphorylation of Bim could be dysregulated in the variants, preventing its ability to induce apoptosis, despite the demonstrated increase in protein expression. However, when we tested for a glucocorticoid-induced increase in phosphorylation of Bim_EL_, we found the increase in both the parental WEHI7.2 cells and resistant variants (CAT2, CAT38, 200R) (Figure 4B). As seen above with resistance to tBid-induced cytochrome *c* release, these data are consistent with a blunted mitochondrial response to BH3-only family members being a source of glucocorticoid resistance in the WEHI7.2 variants.

### 2.4. Cardiolipin Oxidation Is Delayed after Dexamethasone Treatment of the Oxidative Stress-Resistant Variants

**Figure 5:**
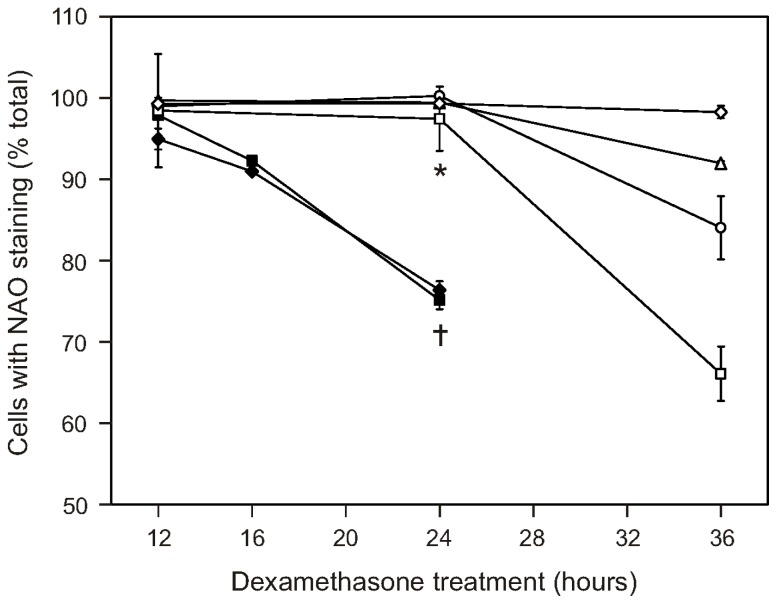
Cardiolipin oxidation is delayed or blocked following dexamethasone treatment of the WEHI7.2 variants. Dexamethasone (1 μM) was added to the cultures at time = 0. At the indicated times, cell samples were collected, stained with 10-N-nonyl acridine orange (NAO) and analyzed by flow cytometry. Cell variants are as follows: WEHI7.2 parental (◆); NEO (■); 200R (□); CAT38 (○); CAT2 (Δ); Hb12 (⋄). The indicated values are relative to the vehicle (EtOH)-treated cultures and represent the mean ± S.E.M. (*n* = 3). * Denotes significant difference between variants and WEHI7.2 cells at 24 h (*p* < 0.05). … Denotes significant difference between WEHI7.2 cells at 24 and 12 h (*p* < 0.05).

Our previous work shows that CAT2, CAT38, and 200R cells remove enough of the hydrogen peroxide generated by dexamethasone treatment to prevent oxidation of the intracellular redox environment [20]. Prevention of cardiolipin oxidation may, therefore, explain the apoptosis resistance seen in the oxidative stress-resistant cells’ mitochondria. To test this possibility, we stained the cells with 10-N-nonyl acridine orange (NAO) at various timepoints after dexamethasone treatment. This fluorescent probe is used to monitor cardiolipin oxidation, based on its binding to the reduced, but not the oxidized, form of cardiolipin [36]. The results are shown in Figure 5. The plotted data points were corrected for the staining seen in vehicle-treated cells, so that the decrease due to dexamethasone treatment can be compared across the cell variants. The plot shows that the extent of cardiolipin staining in the dexamethasone-treated cells closely paralleled the pattern of apoptosis resistance previously observed in our studies of whole cells [17–19]. Within 24 h of dexamethasone treatment, there were significantly fewer WEHI7.2 parental and NEO cells staining positive for cardiolipin. After 36 h of dexamethasone treatment, the percentage of 200R and CAT38 cells with cardiolipin staining was decreased, while CAT2 cells and Hb12 cells show no change in NAO staining. Other investigators have reported that apoptosis of sympathetic neurons due to nerve growth factor withdrawal results in the loss of cardiolipin content, with a consequent decrease in the percentage of NAO-positive staining cells [37]. Our NAO staining results are not explained by a loss of cardiolipin since our previous NMR analyses also show that dexamethasone treatment of WEHI7.2, CAT38 or Hb12 cells does not decrease cardiolipin content [38]; indeed, the cardiolipin content of WEHI7.2 cells increases during apoptosis. A careful study of NAO as a probe for cardiolipin found that factors that alter the spatial arrangement of cardiolipin in the membrane change NAO fluorescence [39]. Oxidation of cardiolipin would be expected to change its spatial arrangement in the mitochondrial membrane.

### 2.5. Discussion

Our data show that the mitochondrial process of cytochrome *c* release is attenuated or blocked at several points in the WEHI7.2 oxidative stress-resistant variants. Selection for resistance to oxidative stress has blunted the dexamethasone-induced signal that causes mitochondria to release cytochrome *c*. Dexamethasone-induced cytochrome *c* release is inhibited at two steps in the mitochondria, untethering of cytochrome *c* from the inner mitochondrial membrane and permeability of the outer membrane. Resistance to both steps of cytochrome *c* release is also in part intrinsic to the variant cells since it is seen following treatment with tBid alone. The data are consistent with the multi-step model for cytochrome *c* release in apoptosis, and the control of reactive oxygen species being rate-limiting for cytochrome *c* release.

Cells with increased catalase blunt the dexamethasone-induced signal for cytochrome *c* release. The decreased ability of dexamethasone treatment to sensitize mitochondria to tBid in the catalase-overexpressing cells suggests the mitochondria contain a hydrogen peroxide target critical for cytochrome *c* release. A current model for the mitochondrial pathway to apoptosis (reviewed in [11,40]) involves oxidation reactions that could explain decreased cytochrome *c* release in cells with increased hydrogen peroxide removal. In this model, the reduced form of cytochrome *c* is associated with the electron transport chain. Once oxidized, cytochrome *c* binds tightly to cardiolipin. This interaction leads to partial unfolding of cytochrome *c* followed by a switch in function from serving as an electron carrier to working as a peroxidase. Cardiolipin is a target of this peroxidase reaction. The oxidation of cardiolipin disrupts the interaction of the two molecules and releases cytochrome *c* from the inner membrane. The catalase over-expressing cells (CAT2, CAT38, 200R) have a limited amount of hydrogen peroxide available to serve as a signaling molecule in dexamethasone-treated cells [20]. Cytochrome *c* may thus be kept in a reduced state, favoring its interaction with the electron transport chain. With limited hydrogen peroxide available to serve as substrates, the peroxidase reaction catalyzed by cytochrome *c* would not occur. The ultimate result is that cardiolipin oxidation and the release of cytochrome *c* are prevented. The decreased cardiolipin oxidation we observed in the CAT2, CAT38 and 200R cells in response to dexamethasone is consistent with this model. However, additional upstream events leading to effects on mitochondria could also contribute to the observed differences in the variants.

Mitochondrial outer membrane permeabilization is another redox-responsive process that would be affected by a decreased hydrogen peroxide signal. Bak and Bax are responsible for forming the pores through which cytochrome *c* is released during glucocorticoid-induced apoptosis [14]. Oxidation of the Cys62 residue causes Bax activation and translocation to the mitochondria [41,42]. Bid-induced mitochondrial membrane permeabilization also requires propagation of an ROS signal [43]. Our data are consistent with both the untethering of cytochrome *c* and the outer membrane permeabilization step requiring oxidants. The relative contribution of each of these oxidation steps to cytochrome *c* release during glucocorticoid-induced apoptosis remains to be elucidated.

Unexpectedly, we found that cells with increased catalase have mitochondria that are intrinsically resistant to apoptosis. Overexpression of catalase by transfection or increasing catalase by selecting for hydrogen peroxide resistance resulted in intrinsic resistance proportional to catalase levels. The resistance is not explained by alterations in the baseline pro- *vs*. anti-apoptotic Bcl-2 family members. We have also ruled out the possibility that increasing catalase simply decreases the baseline hydrogen peroxide in the cells [19,20]. This suggests that increasing catalase altered the mitochondria such that the response to oxidants or tBid is diminished.

Our previous studies of the oxidative stress-resistant WEHI7.2 variants point to mitochondrial modifications that may contribute to intrinsic apoptosis signaling and oxidant resistance. CAT2, CAT38 and 200R cells all have elevated cytochrome *c* compared to the control cells [44]. Increased cytochrome *c* could contribute to the ability to maintain mitochondrial function under stress conditions. Maintenance of mitochondrial electron transport chain function protects against apoptosis [45,46] and oxidative stress [47]. Another possibility is that increasing catalase alters iron metabolism in the variant cells because catalase is a heme protein. Preliminary data from the WEHI7.2 and CAT cells indicates that mitochondrial ferritin is higher in the WEHI7.2 mitochondria (data not shown). Higher mitochondrial ferritin is expected to result in higher mitochondrial iron load and a corresponding decrease in cytosolic iron [48]. Increased expression of mitochondrial ferritin is protective in disease states with dysregulated iron metabolism [49]. However, overexpression of mitochondrial ferritin can increase basal levels of apoptosis [50] and sensitize cells to oxidants [51] in a cell-type specific manner [48]. Thus, it is possible that increased ferritin in the WEHI7.2 cells makes them more sensitive to oxidants and to apoptosis in general; the oxidative stress-resistant variants, with increased catalase acting as a sink for iron, would have less ferritin and be less sensitive to oxidants and apoptosis.

The observation of intrinsic resistance in the oxidative stress-resistant variants has implications for treatment. As in the model of mitochondrial priming proposed by Letai *et al.* [23], BH3-only initiators of mitochondrial apoptosis would be less effective in the variants because of the intrinsic resistance even though the induction is the same. The intrinsic resistance could explain the generalized chemoresistance that we have demonstrated [20], especially to drugs that have no known ROS component. Furthermore, these results suggest that drugs that depend on the intrinsic apoptotic pathway would not be as effective in lymphomas that arise at sites of chronic inflammation.

## 3. Experimental Section

### 3.1. Cell Culture and Reagents

WEHI7.2 murine thymic lymphoma tissue culture cells [52] were grown in Dulbecco’s Modified Eagle Medium-low glucose (Invitrogen, USA) supplemented with 10% bovine calf serum (Hyclone, USA) at 37 °C with 5% CO_2_, in a humidified environment. Cells in suspension were maintained in exponential growth between 2 × 10^4^ cells/mL and 2 × 10^6^ cells/mL. WEHI7.2 cell transfectants overexpressing human Bcl-2 [24] or rat catalase [17], or selected for resistance to 200 μM hydrogen peroxide [18] were maintained as previously described [53]. Cells were subcultured in the absence of selection drug for one week prior to experiments. For dexamethasone-induced apoptosis experiments, cells were diluted to 0.5 × 10^6^ cells/mL and treated with dexamethasone (Sigma-Aldrich, USA) at a final concentration of 1 μM in an ethanol (EtOH) vehicle; control cells were treated with an equivalent amount of EtOH (final concentration = 0.01%). All reagents were from Sigma-Aldrich unless otherwise noted.

### 3.2. Preparation of Recombinant tBid

Recombinant murine p15 tBid (amino acids 60–195) was expressed with a 6-His tag at the N-terminus using the pET23dwHis vector, provided by XM Yin [25]. BL21(DE3) bacteria were transformed with the vector and cultured at 37 °C in Terrific Broth with ampicillin. Expression was induced at 0.6–0.8 A_600_ with 0.4 mM isopropyl β-d-1-thiogalactopyranoside overnight at 18 °C, and bacteria were harvested. Bacterial pellets were resuspended and His-tBid was isolated using the His-Bind Purification Kit (EMD Biosciences, USA) according to the manufacturer’s instructions. Isolated His-tBid protein was concentrated and washed into phosphate-buffered saline (PBS) using a Centricon-10 column (Millipore, USA).

### 3.3. Isolation of Mitochondria

Mitochondria were isolated using a variation on the method published by Wang *et al.* [54]. For dexamethasone-induced apoptosis experiments, cells were harvested after 12 h of treatment with dexamethasone or vehicle. All steps were performed at 4 °C. Briefly, cells were harvested by centrifugation, washed in PBS, and resuspended in mitochondrial isolation buffer (MIB): 10 mM 3-(*N*-morpholino)propanesulfonic acid, 1 mM EDTA, 4 mM KH_2_PO_4_ (final concentration after sucrose addition) at 120 μL/10^8^ cells. After a 30-min incubation on ice, sucrose was added (final sucrose concentration = 0.3 M) and cells were homogenized in a tight-fitting Dounce homogenizer. Nuclei, large debris, and intact cells were removed by centrifugation at 750× *g* for 10 min. The resulting supernatant was centrifuged at 21,000× *g* for 15 min; the mitochondrial pellet was resuspended in MIB including sucrose at 40–50 μL/10^8^ cells, and bovine serum albumin was added to a final concentration of 1 mg/mL. The BCA Protein Assay Kit (Pierce, USA) was used to measure protein concentration of isolated mitochondria. Isolated mitochondria were then used in cytochrome *c* release assays, or SDS-PAGE sample buffer was added and samples were resolved by SDS-PAGE and probed for mitochondrial protein expression using immunoblots, as described below.

### 3.4. tBid-Induced Cytochrome *c* Release Assay

Isolated mitochondria (100 μg) were incubated with recombinant tBid at varying concentrations in a 30 μL reaction in MIB with sucrose at 30 °C for 30 min. Samples were centrifuged at 4 °C, 4,000× *g* for 5 min. The mitochondrial pellet and supernatant fractions were separated, SDS-PAGE sample buffer was added to each, and the samples were analyzed by immunoblotting, as described below. A representative immunoblot from 3–10 independent experiments for each cell type is shown.

### 3.5. Cardiolipin Staining

The percentage of live cells with cardiolipin staining was measured by calculating the percentage of cells in the culture that bound 10-*N*-nonyl acridine orange (NAO) [36]. Following 12, 16, 24 or 36 h of dexamethasone or vehicle treatment, cell cultures were incubated for 30 min in the presence of 100 nM NAO (Molecular Probes, USA) and 5 μg/mL propidium iodide. Cellular fluorescence was measured and analyzed using a FACscan flow cytometer with CELLQuest software (Becton Dickenson, USA). A minimum of three independent experiments were performed and fluorescence from a minimum of 10,000 cells per culture was measured. Only cells that excluded propidium iodide were included in the analysis. Mean percentages of cells with cardiolipin staining were compared using a Student’s *t*-test.

### 3.6. Immunoblot Analysis

Whole cell lysates (25 μg or 100 μg as noted) or isolated mitochondrial fractions (50 μg) were resolved by SDS-PAGE and transferred to polyvinylidene difluoride membranes using standard protocols. Membranes were blocked in 5% milk/Tris-buffered saline with Tween (TBST, 10 mM Tris, 100 mM NaCl, 0.1% Tween-20) for 1 h at room temperature, then incubated in primary antibody in 5% milk/TBST for 1 h at room temperature or overnight at 4 °C. Membranes were incubated in horseradish peroxidase (HRP)-conjugated secondary antibody in 5% milk/TBST for 1 h at room temperature, and proteins were detected by chemiluminescence (Perkin Elmer, USA). To visualize multiple antibodies on the same blot, blots were stripped with Restore Western Blot Stripping Buffer (Pierce, USA), and the above protocol was repeated with the next antibody. The following primary antibody dilutions were used: anti-Bid, 1:500; anti-adenylate kinse-2 (AK-2), anti-Mcl-1, and anti-Puma, 1:1,000 (all from Abcam, USA); anti-β actin, 1:500 (Sigma); anti-second mitochondrial-derived activator of caspase (Smac) and anti-Bim, 1:1,000; anti-Bax and anti-Bcl-x, 1:2,000; anti-Bak, 1:4,000; anti-Bcl-2, 1:2,000, and anti-cytochrome *c*, 1:500–1:1,000 (all from BD Biosciences); anti-phosphoBim, 1:1,000 (Cell Signaling); and anti-heat shock protein 60 (HSP60), 1:40,000 (Assay Designs, USA). The HRP-conjugated secondary antibody dilutions used were: goat anti-mouse-HRP, 1:2,500–1:5,000 (Pierce, USA); and goat anti-rabbit-HRP, 1:2,000–10,000 (Cell Signaling Technology, USA).

## 4. Conclusions

This study demonstrates that selection for resistance to oxidative stress has led to fundamental mitochondrial changes affecting apoptosis. These mitochondrial alterations could be playing a role in the development of lymphoma and resistance to therapy. In the model of glucocorticoid-induced apoptosis used in this study, mitochondria from oxidative stress-resistant lymphoma variants demonstrated resistance to cytochrome *c* release, both before and after treatment with dexamethasone. The variants also demonstrated a delay in the oxidation of cardiolipin following dexamethasone treatment; this is a critical event for cytochrome *c* release. Notably, the observed differences were all proportionate to the degree of resistance to dexamethasone-induced apoptosis previously described for the intact variant cells. Our results provide strong evidence for redox regulation at several points in the mitochondrial pathway to apoptosis. It is noteworthy that similar changes are found in mitochondria from lymphoma cells made resistant to oxidative stress by different approaches (catalase transfection and selection by hydrogen peroxide treatment). The results from this model system may, therefore, be applicable to other cases of oxidative stress-adapted cancer cells. Letai and colleagues have shown that ‘primed’ tumors, *i.e.*, those that are more sensitive to mitochondrial outer membrane permeabilization, are closer to the apoptotic threshold, and exhibit a superior clinical response, compared to poorly primed tumors [23]. In light of data presented here, cancers arising in settings of chronic inflammation may be particularly resistant to chemotherapeutic agents that work through the intrinsic pathway to apoptosis.

## Supplementary Materials
