# Supplementary material for: Mitochondrial Adaptations to Oxidative Stress Confer Resistance to Apoptosis in Lymphoma Cells
Source: Int J Mol Sci. 2012 Aug 16;13(8):10212–28. doi: 10.3390/ijms130810212 (PMC3431854; doi:10.3390/ijms130810212)

# Supplementary Material

**Figure S1.** Cytochrome *c* and AK-2 release is induced by tBid: vector control. Vector transfected control cells (NEO) were pre-treated with 1 μM dexamethasone (**A**) or EtOH vehicle (**B**) for 12 h. Mitochondria were isolated, treated with various concentrations of recombinant tBid, and separated into mitochondrial (M) and supernatant (S) fractions. Representative immunoblots for cytochrome *c*, AK-2, and HSP60 are shown.

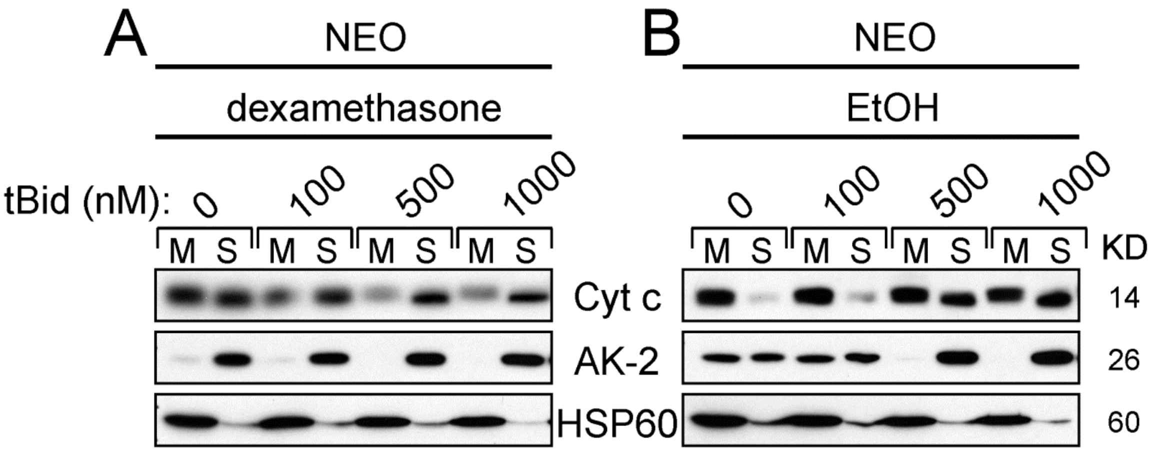


**Figure S2.** Cytochrome *c* and AK-2 release is induced by tBid: HSP60 control for integrity of the inner mitochondrial membrane. WEHI7.2 parental, 200R, CAT38, CAT2, and Hb12 cells were pre-treated with 1 μM dexamethasone (**A**) or EtOH vehicle (**B**) for 12 h. Mitochondria were isolated, treated with various concentrations of recombinant tBid, and separated into mitochondrial (M) and supernatant (S) fractions.

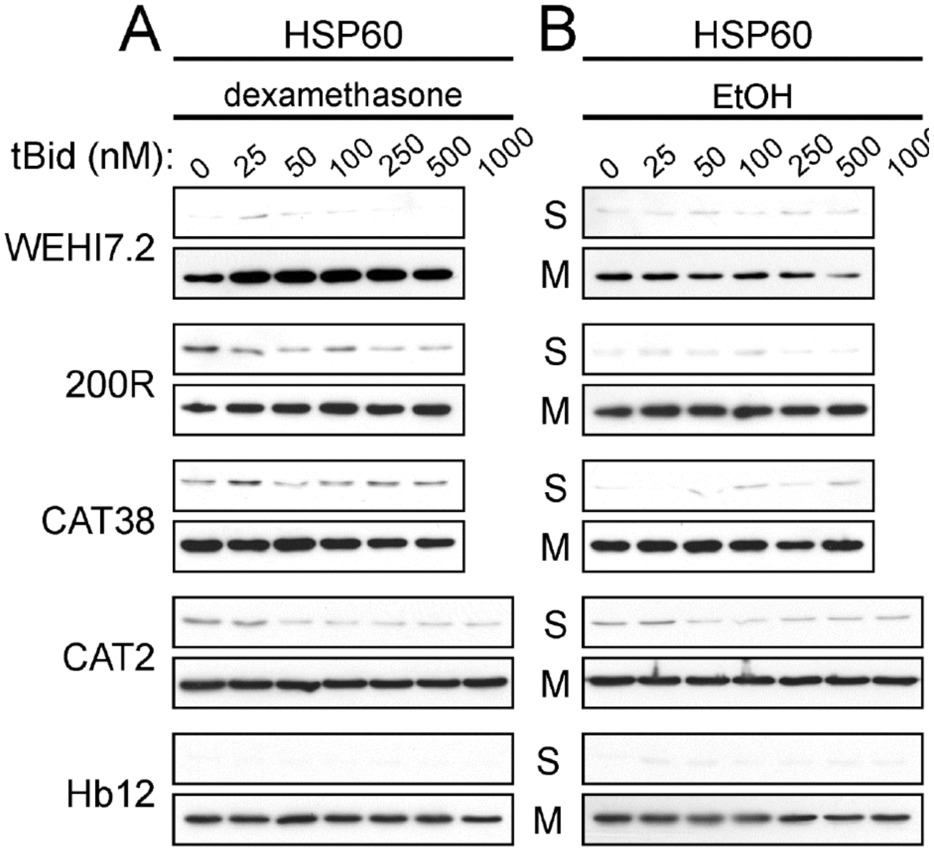

**Figure S3.** Smac/DIABLO release is induced by tBid. WEHI7.2 parental, 200R, CAT38, CAT2, and Hb12 cells were pre-treated with 1 μM dexamethasone (**A**) or EtOH vehicle (**B**) for 12 h. Mitochondria were isolated, treated with various concentrations of recombinant tBid, and separated into mitochondrial (M) and supernatant (S) fractions.

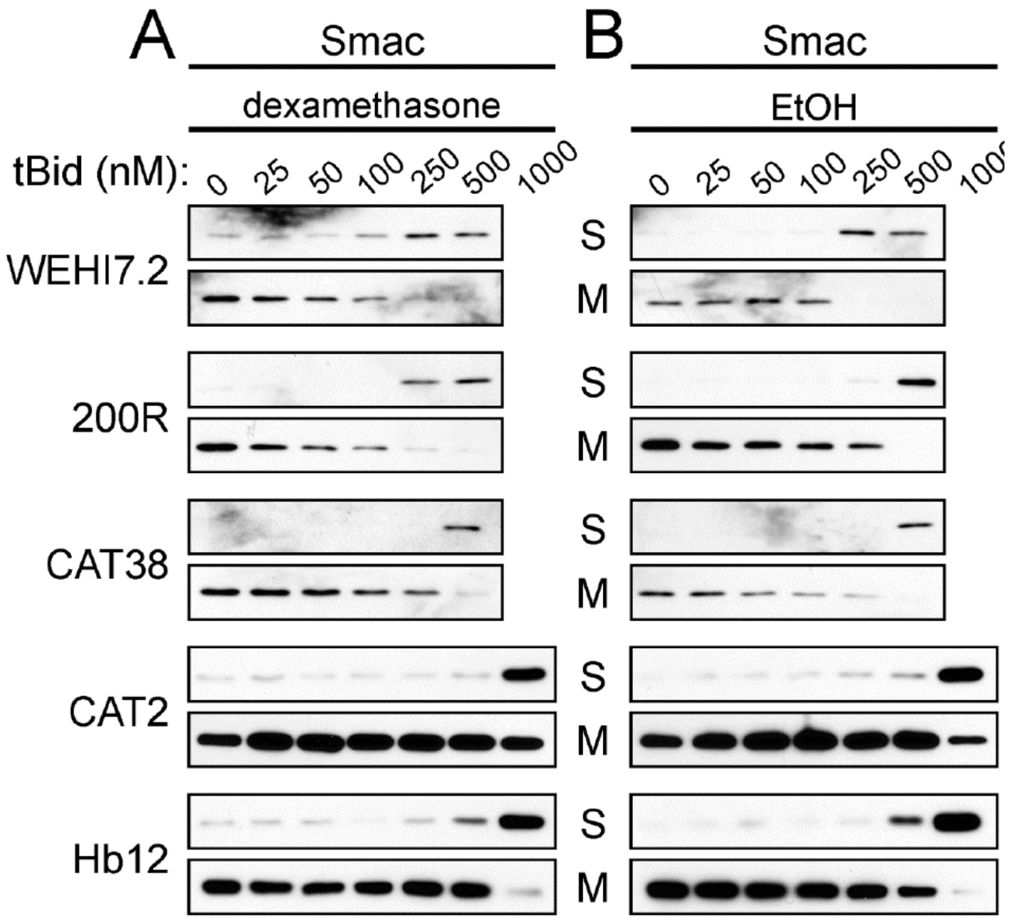

Supplement: Supplementary file 1 [file ijms-13-10212-s001.pdf]
